# Private Surgical Practice

**Published:** 1877-04

**Authors:** Jno. E. Owens


### PRIVATE SURGICAL PRACTICE.

### CASES OF INTERNAL HÆMORRHOIDS—OPERATIONS BY THE CLAMP AND ACTUAL CAUTERY.

UNDER THE CARE OF JNO. E. OWENS, M. D.

CASE 6. Mrs. ——, aged 47 years, came under my care for internal hæmorrhoids, March, 1874. She is the mother of nine children, the last of which was born in September, 1872, and lived but a few days. This lady, in consequence of the rectal disease, had been for some years an invalid, and totally unable to attend to her household duties. Exhausting pain and hemorrhages rendered life itself burdensome. Frequently overwhelmed with gloomy forebodings, and always exhausted, she was absolutely compelled to withdraw from the society of a very large circle of acquaintances. Having had the professional care of herself and family for a considerable time, I took advantage of many opportunities during the last two years of her suffering, to induce her to submit to an operation for the radical cure of her disease. After very many so-called cures had failed, as they always do in such cases, she submitted to the operation. The first symptoms, preceded by a troublesome constipation, occurred about fifteen years ago, two months after the birth of a child. Her parturition on this occasion was normal, and her "getting up" was good. Exercise and cold increased her suffering. Eleven years ago, the affection became very troublesome. Even a moderate walk was sufficient to bring on "an attack of piles." During the last eight years, hemorrhages which, prior to this period, had been moderate, became at times profuse, the loss of a teacupful of blood having been of common occurrence. Eighteen or nineteen years ago, a period began and extended over eight years, during which she did not menstruate—doubtless owing to pregnancy and lactation. Four children, born during this time, were nursed as follows: One, eighteen months; one, one year; and the third and fourth, two years each.

A few external hæmorrhoids were the first to attract her attention. These inflamed and ulcerated many times, and when in this state, caused even more suffering than the internal piles. The latter always came down after stool, but usually returned without difficulty. There was also some prolapse of the rectum. Pain and soreness were constant. The former, located for the most part, in the lower part of the back, was more intense about the rectum, but extended somewhat towards the front. Constipation was not troublesome during the last five years, owing to the ability of the patient to guard against it. The loss of blood was a serious drain upon the patient's vital powers. After an attack the eyes were weak and tearful. The patient looked much older than she really was, and spent much of her time in bed. One of the internal tumors, called "perineal," on account of its being located in the anterior segment of the rectum, contiguous to the perineum, was the most troublesome to the patient. It was always the first to prolapse; it was frequently the only one that came out, and was, from its location when out, the most closely encroached upon by neighboring parts. From such causes, this variety of pile is generally the most painful. Ether having been administered the clamp and cautery were applied to four internal hæmorrhoidal tumors, March 16, 1874. Several external piles were removed with scissors, and the sphincter muscle was "overdistended."

March 18. The swollen mucous membrane began to roll from the anus, and on March 20th, it had reached its acme, being about the size of an English walnut. Pain on 18th and 20th called for morphia.

March 21. Alvine evacuation in response to syr. senn. comps. [1] and an injection of warm sweet-oil.

Cotton saturated with the following lotion was applied to the swollen mucous membrane: Ext. belladonnæ, grs. iv.; ext. opii, grs. vj.; liq. plumbi subacet. dil, i℥.

March 25. Following an evacuation, the pain continued from one to two hours. After trying various laxatives, I found the following to act quite comfortably: Ext. colocynth, ext. hyocyam. āā grs. xvii.; ext. nucis vom, grs. iii.; Make 12 pills. S. one, two or three times daily, if necessary. Injections of warm sweet oil were advantageous.

March 29. Bowels move without pain.

March 30. Bowels move without the aid of medicine; patient up to-day—two weeks from the day of the operation; liberal cold water bathing of the anus, and when necessary a warm water enema.

March 31. Menstruating.

April 2. Able to ride out.

April 7. Rode out; patient is free from all discomfort, and expressed herself as feeling better than she had done for a number of years.

March 1, 1877. Patient is perfectly well at this date.

Case 7. Mr. ——, aged 35 years, had suffered from internal hæmorrhoids for nine years. Prof. De Laskie Miller, the family physician of this gentleman, referred him to me for operation. The patient is of the opinion that the hæmorrhoids were the result of straining at stool, having been unwilling to spare sufficient time from business for defecation. During the last three years of their existence, the piles, together with a portion of the rectum, prolapsed at stool, and could only be replaced by pressure and other manipulation. For a month preceding the operation, the pain was excessive, and it often required upwards of a half hour to return the protruded mass. During his efforts at replacement, the patient found it necessary

1. One part fluid ex. senna, three parts syrup.

to assume various positions, namely: on his back, on one side or the other, the legs and pelvis elevated, etc. Certain days he was obliged to leave his business from twenty to twenty-five times, in order to effect a reduction of this bleeding and painful protrusions. Pain was, more or less, severe for four or five years, but he had hemorrhage frequently during nine years; three years before the operation the blood began to run in a stream; at times he noticed several streams; more lately, he had hemorrhages daily for five or six days. Two week's freedom from hemorrhage was a rare occurrence. After some of his hemorrhages, the pulse beat reached 120. Being a man of much pluck, however, he put in a daily appearance at his place of business. He was frequently annoyed by cramps in his intestines; a sense of fullness in the top of the head, and a peculiar sensation in the eyes, almost invariably foreshadowed "an attack" and a hemorrhage. The protruded mass, frequently equal in size to half the first, grasped by the sphincter muscles, was productive of an agony, from which the patient was eager to be relieved at any cost. "An attack" was almost invariably the penalty paid for an indulgence in alcoholic stimulants. Anæmia and debility were prominent symptoms.

OPERATION.—The patient having been ætherized, Sept. 25, 1875, I made seven applications of the clamp and actual cautery. The part was very generally hemorrhagic, and bled freely, even upon careful manipulation. The mucous membrane in patches presented a granular appearance, and in addition to the larger tumors, small granular masses gave out even upon puncture a liberal and persistent flow of bright arterial blood. These latter are described as capillary hæmorrhoids. Two of the tumors, together with the redundant mucous membrane, were so large at their pedicles as to extend somewhat beyond a clamp of the largest size. After dealing with the tumors, other portions of diseased surface that bled was seared. One of the larger tumors was quite solid, and creaked when cut with scissors. A dose of deodorized ti. opium (grs. xx.) was ordered by Dr. Miller. The patient was much annoyed, and was kept awake by a frequent and painful contraction of the sphincter. All hemorrhage had ceased when the rectum was returned.

HEMORRHAGE.—In two hours after the completion of the operation, I was summoned to see the patient. Nauseated by the æther, he had been vomiting; straining to move the bowels and tossing over the bed in a more or less violent manner. He was under the impression that the bowels had moved, but clots and fluid blood in large quantities had been voided. The bed clothing having absorbed the fluids discharged, the patient was covered with blood from head to foot. I sat by him and introduced ice suppositories for two hours. There was no return of the bleeding.

There was suppression of urine for thirty-six hours after the operation; the sixth day after operation, bowels moved copiously in response to castor oil.

From two to four grains of opium daily were required to allay pain, and to produce constipation.

Oct. 3. Bowels moved with but very little pain, in response to "bitter-water."

Oct. 4. Patient up to-day, about twenty minutes; instructed to secure a daily evacuation of the bowels by injection; patient up every day from this date.

Went down stairs on the 18th day; went out the 20th day; went to business on the 22d day, to remain a few hours.

REMARKS.—The clamp should be used so that the tissues to be cauterized shall not project beyond the end of the clamp. In this operation, the tissues of two of the tumors thus projected, and this, together with the straining, and other violence above referred to, was probably the cause of the hemorrhage. The sphincter muscle was not overdistended in this case, and hence the annoyance occasioned by its frequent spasmodic action. Even liberal doses of opium were insufficient to entirely avert this painful spasm. The more the parts contained within the grasp of the muscle are swollen, the more do they encroach upon the muscle, which resents such encroachment by painful contractions. By thoroughly "overdistending" the muscle at the time of the operation for the hæmorrhoids, this source of suffering may be avoided.

Patient is perfectly well at this date, March 12, 1877.

